# Integrating HIV prevention with family planning services for adolescent girls and young women in Uganda: perspectives of adolescent girls and young women, health care providers, and policymakers

**DOI:** 10.3389/frph.2025.1441829

**Published:** 2025-02-24

**Authors:** Justine K. Tumusiime, Davina Canagasabey, Peter Mudiope, Sabrina Kitaka, Allen Namagembe, Fiona Walugembe, Bridget Nakankaka, Josephine Nabuuma, Jesca Nasunku, Jane Cover, Betty Mirembe, Ashley Jackson, Kimberly Green

**Affiliations:** ^1^ PATH, Uganda Country Program, Kampala, Uganda; ^2^ PATH, Primary Health Care, Washington, DC, United States; ^3^ AIDS Control Program, Ministry of Health, Kampala, Uganda; ^4^ Pediatrics and Child Health Kampala, Makerere University College of Health Sciences, Kampala, Uganda; ^5^ AGYW Peer Researcher, Masaka, Uganda; ^6^ AGYW Peer Researcher, Nakasongola, Uganda; ^7^ PATH, Primary Health Care, Seattle, WA, United States; ^8^ PATH, Primary Health Care, Geneva, Switzerland

**Keywords:** HIV, family planning, AGYW, PrEP, integration, prevention, human-centered design (HCD), Uganda

## Abstract

**Introduction:**

Persistently high HIV incidence among women, especially adolescent girls and young women (AGYW), have drawn the attention of national policymakers, donors, and implementers in Sub-Saharan Africa to the integration of HIV and family planning (FP) programs. According to several research studies, FP services could offer a holistic strategy to address the HIV and FP needs of this demographic by including HIV prevention approaches, particularly HIV pre-exposure prophylaxis. Our study set out to explore the obstacles and opportunities that AGYW faced in accessing, using, and continuing HIV prevention and contraceptive services; to develop ideas for novel service models that would allow AGYW to receive integrated, HIV prevention and contraception services; and to evaluate the viability, scalability, and acceptability of these models through dialogues with stakeholders using a human-centered design approach.

**Methods:**

We conducted 128 in-depth interviews with 60 AGYW, 24 public and private health care providers, 10 community leaders, and 10 policymakers). We also conducted two co-creation workshops with 50 AGYW and 10 health care providers in Masaka and Nakasongola districts to generate service delivery models.

**Results:**

Our findings reveal various enablers and barriers to the successful integration of HIV prevention into FP services in the areas of policy, human resources and infrastructure, resource management, service delivery, community involvement, supply chain management, and monitoring and reporting.

**Discussion:**

Successful integration will require addressing key concerns raised by participants in human resource and infrastructure, resource management, service delivery, demand creation, male involvement, supply chain management and monitoring and reporting.

## Introduction

In Africa, 25.6 million people were HIV positive in 2022, with 20.8 million of those cases occurring in eastern and southern Africa (1). While the HIV response in eastern and southern Africa has made major progress—including a 59% reduction in annual number of people acquiring HIV—adolescent girls and young women (AGYW) aged 15–24 years remain disproportionately affected, with 27% of new HIV infections occurring among AGYW, and AGYW three times as likely to acquire HIV than their male counterparts (2) Research studies conducted in sub-Saharan Africa indicate that women, particularly young women, are at increased vulnerability to HIV compared to men (3, 4).

The World Health Organization (WHO) recommended inclusion of daily oral pre-exposure prophylaxis (PrEP) as part of a combination prevention approach for people with substantial vulnerability to HIV in 2015 (5). Despite its effectiveness and potential as a critical tool for preventing acquisition of HIV, PrEP use remains sub-optimal and below 2025 prevention targets across most regions, including eastern and southern Africa (6). This is in part due to challenges delivering safe HIV prevention and contraception services in Africa, given obstacles such as ineffective translation of policy into actionable implementation plans, inadequate provider training impacting quality of care and counseling, and limited community involvement driving lower awareness and demand (7). Stigma associated with seeking PrEP at a health care facility as well as privacy concerns serve as a significant deterrent to effective PrEP use and adherence among AGYW (8, 9). Other obstacles to PrEP implementation, initiation, and persistence encompass stigma associated with using antiretroviral-based products, adverse drug effects, relocation difficulties, insufficient resources for screening and monitoring, and a scarcity of qualified health care workers for PrEP distribution (10).

Data from the Evidence for Contraceptive Options and HIV Outcomes project shows that 4.3% of AGYW who visited family planning (FP) clinics were HIV positive (11). Studies have also observed that sub-Saharan Africa, as a region with the highest HIV prevalence, also has the highest prevalence of unmet need for contraception (12). These findings prompted WHO and other normative agencies to advocate for the inclusion of HIV prevention, including PrEP, in FP programs, especially for AGYW living in high-prevalence areas (13). A factor driving this endorsement is that women seeking FP services have similar vulnerabilities for HIV, sexually transmitted infections, and unintended pregnancies. Integrating a comprehensive range of sexual and reproductive health (SRH) services, including PrEP provision, within FP clinics could be both feasible and advantageous (14). Integrated service delivery has been globally recognized as a strategy to improve access to and acceptance of comprehensive HIV prevention and FP services (12). Integrating HIV prevention initiatives into FP services provides a promising approach to enhancing access to and use of HIV prevention services among women, particularly AGYW, while also maximizing the existing infrastructure for delivering comprehensive care (15). Through the integration of HIV prevention initiatives into FP services, programs can synchronize with the current practices and preferences of clients, tailoring interventions to more effectively meet the needs of AGYW seeking both types of services (16). Integrating HIV prevention services into the FP service platform also has the potential to address PrEP stigma in clinic settings, if stigma reduction interventions, including provider sensitization, are identified and applied (8).

Despite the advantages of integration of HIV prevention and FP services, some studies have reported challenges including staff turnover, insufficient planning, budgeting for training, and low demand for certain services like intrauterine devices (IUDs) (12). These challenges can lead to gaps in service provision and diminish the effectiveness of integrated care models. Additionally, the integration of services may face other obstacles related to space, time, and shortages of essential commodities, as reported by providers delivering integrated care in various settings (17). Other studies also point to inadequate resources, including physical space, consultation time, trained staff, and essential commodities as factors that could impede the seamless delivery of integrated services and compromise the quality of care provided to clients seeking both HIV prevention and FP services. Related to staff training, difficulties in dual training and supervision of providers due to the chronic shortage of health personnel in sub-Saharan Africa are also barriers to the effective integration of services (18).

The evidence on integration of PrEP into FP services in sub-Saharan settings indicates it is feasible if considerations are made to address identified staffing, training, commodity management, and resource-related barriers, yet the uptake of PrEP by screened and eligible AGYW is low (16). As such, it is crucial to understand clients' viewpoints on challenges and opportunities concerning the uptake, effective use, and continuation of HIV prevention and contraceptive services; develop concepts for innovative service models to deliver voluntary, integrated HIV prevention and FP services; and evaluate the feasibility, scalability, and acceptability of these models through dialogues with stakeholders.

## Materials and methods

### Study overview

We led research in two districts of Uganda to inform development of feasible, acceptable, and scalable models for integrating HIV prevention into FP services for AGYW aged 18–24 years. Applying PATH's human-centered design Living Labs methodology, we specifically aimed to gather information across three main objectives: (1) AGYW perspectives on opportunities and challenges related to uptake and continued effective use of integrated HIV prevention and FP services; (2) service delivery models offering integrated HIV prevention and FP services for AGYW; (3) assessment of feasibility, acceptability, and scalability of recommended service delivery models by key stakeholders.

Study districts were selected in collaboration with the Uganda Ministry of Health (MOH) to ensure geographic and service coverage diversity. The two selected districts were:

• Masaka, an urban district with existing Determined, Resilient, Empowered, AIDS Free, Mentored, and Safe (DREAMS) programming supported by the U.S. President's Emergency Plan for AIDS Relief (PEPFAR), chosen for its higher awareness of and experience with FP and HIV prevention services.
• Nakasongola, a rural district without existing DREAMS programming, selected to represent a population potentially less experienced with HIV prevention and FP services.

### Research team

Research was led by four AGYW peer researchers, with support from experienced local research assistants and a study team at PATH. We identified and recruited four AGYW (two per study district) aged 18–24 years working as peer supervisors in DREAMS safe spaces in Masaka district or adolescent clinics in Nakasongola district to be peer researchers, with input from health care providers at Masaka DREAMS safe spaces and Nakasongola adolescent clinics. The four AGYW peer researchers were supported through ongoing coaching and mentoring by senior research assistants. Both peer researchers and senior research assistants were trained on study protocol procedures, qualitative data collection techniques, participant consent requirements and processes, data cleaning, transcription, and data upload. They were also trained in research ethics, the Living Labs human-centered design approach, and communication skills to facilitate interviews and mobilize participants.

To ensure alignment with global and national policy and community preferences, PATH, in coordination with Uganda MOH, convened a small group of subject matter experts in two technical advisory groups (TAGs)—a national TAG within Uganda and a TAG at the global level. At the global level, we prioritized global leaders in biomedical HIV prevention, FP, adolescent health, and service integration, including representatives from major donors (e.g., PEPFAR, the Bill & Melinda Gates Foundation, the Children's Investment Fund Foundation, the Global Fund to Fight AIDS, Tuberculosis, and Malaria, the United Nations Agency for Children, and the United Nations Population Fund), WHO, and implementers. TAG members were convened on a quarterly basis in-person (national TAG) and virtually (global TAG) to hear updates from the study team, provide input on research implementation and analyses (e.g., study protocol development, results interpretation), weigh in on applicability of findings, and provide overall oversight of study implementation.

### Study population and participant selection

Five groups of participants were engaged in the study, with all participants required to be at least 18 years of age (with the exception of emancipated AGYW) and having provided informed consent for participation in our study:

1. HIV/FP and health system stakeholders: These included national and subnational policymakers and other health system stakeholders involved in formulating, implementing, and/or enforcing adolescents health policies in Uganda. Subnational stakeholders had to either reside or work in either study district at time of data collection.
2. Health care providers: This group included facility, community, or peer health care workers in either private- or public-sector settings involved in providing HIV prevention or FP services to AGYW.
3. Community leaders: These include influential community personnel residing or working with youth or/and youth-related local groups and organizations in either of the study districts, focusing on enabling youth make the best use of their skills, talents, expertise, and imagination.
4. AGYW: Adolescent girls and women aged 18–24 years or emancipated minors (heads of family or holding primary responsibility for their care/well-being; pregnant and/or has children; cohabiting or married) below 18 years residing in one of the two study districts at time of study participation. Other eligibility criteria was also a requirement to have used HIV prevention and FP services before.
5. Health facility in-charges: Heads of private- or public-sector health facilities in Masaka or Nakasongola district at time of study participation.

We used a convenience/purposive sampling and recruitment process. The study team worked with Masaka and Nakasongola district health teams to select public- and private-sector facilities, among which health care workers and facility in-charges were selected for potential study participation. District health teams also identified community leaders and policymakers for potential study participation. AGYW accessing HIV prevention and FP services at proposed health facilities during clinic days were identified for potential study participation. Refreshments were provided during in-depth interviews (IDIs), focus group discussions (FGDs), and workshops, and participants were provided with stipends to cover transportation to IDI, FGD, and/or co-creation workshop venues; no additional incentives were provided.

### Data collection

Using a user-centered and peer-led research approach, we engaged 128 participants in IDIs—60 AGYW, 24 public and private health care providers, 24 public and private health facility managers/in-charges, ten community leaders, and ten policymakers. We conducted four FGDs—two with AGYW and two with health care providers in each district. We held two face-to-face co-creation workshops, one in each study district, with 50 AGYW (25 per district) and 10 health care providers (5 per district) to ascertain preferences for integrated HIV prevention and FP service delivery models and understand AGYW HIV prevention and FP service access constraints. FGDs and IDIs for AGYW were conducted in Luganda, the predominant local language in both study districts, while other interviews were conducted in English and/or Luganda, based on participant preferences. Co-creation workshops were conducted in Luganda for AGYW and in Luganda/English for health care providers.

All FGDs and IDIs were recorded digitally and transcribed directly into English, utilizing meaning-based translation from Luganda where necessary. Co-creation workshops were audio recorded, with recordings were transcribed directly into English language. All transcripts were cross-checked for accuracy against the original recordings and field notes by the study team, with backup files stored after each transcription session. Miro was used as a designated data collection system.

### Data analysis

Transcripts were then uploaded to QSR NVivo 14 qualitative analysis software for coding by a qualitative analyst. The qualitative analyst employed thematic analysis, involving the identification of explicit and implicit ideas in the data and labelling these ideas as themes across the dataset. This process included the following stages: (1) familiarization with the data by reading all transcripts; (2) generation of initial codes emerging from the data and refining the coding framework as more data was coded; (3) extraction of all statements pertinent to identified sub-themes, with verbatim statements coded under relevant themes; and (4) labeling of all statements using QSR Nvivo 14 qualitative data analysis software. This methodology ensured a comprehensive understanding of the data and facilitated the identification of key themes relevant to the research objectives.

## Results

An analysis of findings related to the perspectives of AGYW, policymakers, and health care providers regarding the integration of HIV prevention and FP services was carried out across six major domains: policies, human resources and infrastructure, resource management, service delivery models, HIV prevention and FP services use, and monitoring and reporting. The key findings are presented in sections corresponding with the six domains of focus. For each of these domains we highlight the barriers to and enablers of integration (see Table 1).

**Table 1:** Summary of integration enablers and barriers across the domains.

Domain	Integration Enablers	Integration Barriers
Policies	• Existing support of policymakers for integration • Current policy guidelines allow for FP and HIV services to be provided concurrently	• No dedicated resources for integration
Human resources and infrastructure	• No additional staff needed for integration but there is a need for task sharing and additional training support	• Increased follow-up and reporting as well as long waiting time at the health facility as a result of integration • Limited space within health care facilities to deliver integrated services in a private and friendly manner
Resource Management	• Reliable supply of FP and HIV prevention commodities in government health facilities and non-governmental organizations	• Stockouts and limited supply especially of HIV prevention commodities in the private health facilities
Service Delivery	• VHTs could sensitise communities and deliver the integrated services at the village level • Some VHTs have already been trained in the delivery of HIV and FP services • Stocks of HIV prevention and FP can be managed at the health facilities that the VHTs are attached to. • Existing government health centers can be stocked with HIV prevention and FP commodities to enhance access • The private sector (including clinics, pharmacies, and drug shops) and non-governmental organizations can offer integrated HIV prevention and FP services • End users willing to use a voucher system to access the integrated services	• Health care providers in some of the private health facilities, such as drug shops and pharmacies, may require additional training on offering proper counselling to PrEP users, guarding against misuse of drugs, and in following all required steps and clinical examinations before enrolling clients on and/or dispensing PrEP • The cost factor may limit access to integrated HIV prevention and FP services in the private sector. • Knowledge gaps among health care providers to offer the integrated services
Use of integrated HIV prevention and FP services	• Integration seen as a way of fostering confidentiality and privacy by end users. • Integration provides a platform for raising awareness of HIV prevention services • More holistic care can be provided through an integrated model	• Some HIV prevention services like PrEP are not known to many people in the general public • Concerns of prolonged waiting time and overcrowding at the health facility with the integrated services compromising confidentiality • Lack of required equipment and commodities at the service delivery point, for example, the creatinine test needed to be carried out before administering PrEP
Monitoring and reporting	Continuous training and supportive supervision	• Health care providers using different registers to capture information on HIV prevention and FP services • Increased reporting and follow up with the integrated services overwhelming health care providers and compromising the quality of data collected

### Policies

Policymakers, community leaders, and health system managers interviewed in our study supported the integration of HIV prevention and FP services, saying it would minimize duplication of services, increase access to these services, and address issues of stigma, particularly related to PrEP use.

“In terms of logistics management, would it be cost effective for you to say tomorrow the vehicle is bringing contraceptives. The other day another vehicle is bringing condoms. The other day it is delivering ARVs. I think when all these are put and delivered together at a go, in terms of economical aspect of managing delivery and distributions of health commodities, integration is the best approach.” (District Health Officer)

Policymakers highlighted that the Uganda MOH prioritized integration and drafted an update to the national strategy for the integration of SRH, HIV, gender-based violence, tuberculosis, and nutrition (2022–2025), providing strategic guidance for integration at the policy, programming, and service levels. Data tools have been revised, and training and roll out are pending. The strategy also included capacity building of health care providers to deliver integrated services and linkage of clients to entry points where they could access integrated services. The MOH acknowledged that there was need to mobilize financial and human resources to facilitate the delivery of integrated HIV prevention and FP services and acknowledged that this study was timely to inform development and optimization of preferred models.

 “Ministry of Health is already committed to integration of services, strategy is already drafted, it’s not like we are bringing new services onboard but these are services that have been offered for a long time, so we just need to put a few things together.” (Policymaker)

### Human resources and infrastructure

Given the ongoing concerns regarding human resource constraints at health facilities, policymakers, facility in-charges, and health care providers indicated a need to increase staffing levels if integrated HIV prevention and FP services were to be successfully delivered. Importantly, special attention should be paid to certain cadres that are currently not included in PrEP provision, such as community health workers who have done a commendable job in improving AGYW access to FP services. Stakeholders felt that engaging all categories of health workers to deliver integrated services allows for task sharing and could contribute to resolving staffing challenges within health care facilities. Stakeholders did observe the need for comprehensive and continuous supportive supervision for health care providers on integrated delivery, focusing on quality of FP and HIV counseling to clients and recording data in registers.

 “I think integration will work very well looking at the current and maybe the future staffing levels because government is not going to have the resources to employ a person per service. So, to be able to minimize operational costs in terms of employing more human resource, integration becomes the way to go.” (District Health Officer)

Some health care providers noted no need to increase the number of staff at health facilities, but rather that facilities should make some changes internally to enable the provision of integrated services, noting that integration could actually reduce workload. In addition, policymakers and facility in-charges called out a need for health care worker training on both HIV prevention and FP services before they engage in delivery of integrated services. In the future, the country is looking towards incorporating integrated service delivery into pre-service training curriculums to reduce in-service provider training costs.

“What is most important is for the provider in that facility to be trained enough or they may be educated but need training on how those services are offered. When a health care provider is well trained, they will offer the service to the client’s satisfaction and sensitizing them to understand their proper use. A health care provider could offer family planning services to the client without being able to talk to them about HIV prevention. Therefore, it is up to the providers to be well trained to effectively offer these services.” (Facility in-charge from Masaka district)

Health care providers shared concern about an increased workload following the integration of these services, due to increased reporting and follow up. Some facility in-charges and health care providers as well as AGYW were concerned that long waiting times at health facilities to access both services could discourage some clients.

 “Addition to that there is filling in the client’s information in the family planning registry which is a very long detailed information to be written. Then after that, you need to fill in the HIV registry book which is also too much work, then you have to insert the method of family planning more so the long-term methods take some good time while inserting the method and at the same time you have to give HIV prevention methods. So all together it consumes a lot of time and hectic and you will end up spending more than one hour only on a client.” (Health care provider from Nakasongola district)

“One challenge could be the clients might take longer time because you are entering now in two different registers so there is some long time waiting.” (Facility in-charge from Nakasongola district)

Notably, policymakers, health facility in-charges, and health care providers raised challenges regarding the limited space within health care facilities to deliver services privately. For example, some facilities lack structures where a separate space for integrated HIV prevention and FP services could be delivered, such as youth corners for AGYW to comfortably access these services. This lead to some participants calling for investment in new infrastructure to enable delivery of HIV prevention and FP services in a private, confidential, and friendly manner.

### Resource management

Availability of commodities was considered crucial for successful delivery of integrated services. Stockouts of HIV prevention and FP commodities at the health facilities was cited as a big challenge, especially in private health facilities where PrEP was reported to not be available.

** “**The other challenge I anticipate is the commodity supplies; if you are talking about Family Planning, do you have the commodities? If you are talking about HIV prevention, what is the method to offer to this person? So you have to make sure that you don’t frustrate the clients who have come for the services.” (Facility in-charge from Nakasongola district)

Both policymakers and health care providers emphasised the need to ensure that there was a consistent supply of HIV prevention and FP commodities at health facilities offering integrated HIV prevention and FP services. Most health care providers and facility in-charges were of the view that the successful integration of HIV prevention and FP services requires increased commodity supplies to support both services being jointly delivered, as their demand was likely to increase.

“The integration would be good if supplies would come timely. It will help those people on ART to know about family planning existence and if you talk about it, someone can pick interest and start.” (Facility in-charge from Nakasongola district)

Participants in the co-creation workshop said that PrEP and FP commodities at public-sector health facilities could be managed by National Medical Stores (not implementing partners) and delivered to health facilities through the national supply chain. For private-sector facilities, they suggested availing subsidised branded products.

### Service delivery

Several models were suggested for the successful delivery of integrated HIV prevention and FP services, including delivery through public and private sector entities and community-based models leveraging village health teams (VHTs) and other outreach mechanisms.

**Figure 1:**
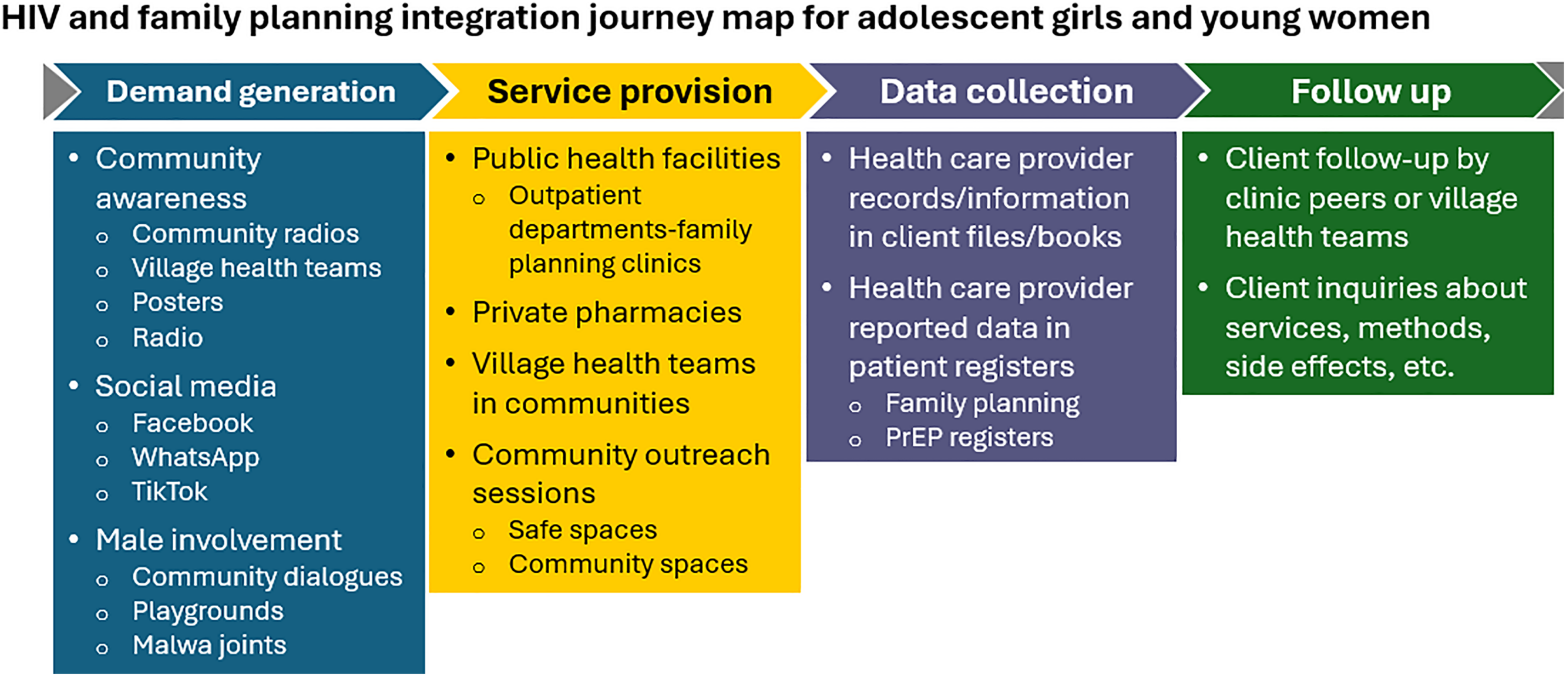
HIV and FP integration journey map for AGYW.

Participants at the co-creation workshop, described the preferred journey for the delivery of the integrated HIV and FP services (see Figure 1). First, demand creation through enhanced awareness-raising in communities, including using social media and including men in mobilization efforts. Next, services could be delivered through various entry points, including public health facilities, private pharmacies, VHTs, and community outreach. Health care workers should collect and record data collection in client books or cards, with ongoing recording of client data in PrEP and FP registers. Follow up of clients could be carried out by clinic peers or VHTs.

#### Offering community-based integrated services through VHTs and outreach sessions

Findings from the interviews were consistent with what emerged from the co-creation workshop. At the grassroot level, health care providers suggested that VHTs be engaged to carry out integrated community sensitization and service delivery, pointing out that it may be difficult for VHT to deliver PrEP although they have already been trained to deliver injectable contraceptive DMPA-SC (Sayana Press) in communities.

**“**I think that is easy. If we can’t get it here, we work with VHTs who are close to the people to sensitize people more and offer some services because currently VHTs are offering Sayana.” (Facility in-charge from Masaka district)

“It [referring to the government] has trained the VHTs on different health related aspects, and now the VHTs can take HIV blood tests from people in the community, and also government has extended the services to the community and the VHTs are really doing their job well, they have even started offering these oral PrEP tabs in the communities.” (Community leader from Nakasongola district)

Health care providers said that VHT members should be trained on how to offer integrated services and recommended they obtain the needed stocks of HIV prevention and FP commodities from health facilities that they are attached to. Community outreaches were also suggested as another channel for extending the reach of integrated HIV prevention and FP *n* services to end users. Overall, participants were of the view that all government health facilities, including health centre IIs (the lowest level of facility-based PHC service delivery), needed to be stocked with HIV prevention and FP commodities to enhance accessibility to users.

“The government should extend the services and bring equipment to these lower-level health facilities (health centre IIs) deep in the villages [so] people in those remote communities can easily access these commodities without travelling long distances to only health centre IVs.” (Community leader from Nakasongola district)

#### Delivering integrated HIV prevention and FP services through the private sector

The private sector was also cited by AGYW, facility in-charges, health care providers, and community leaders as another crucial strategy for ensuring last-mile delivery of integrated HIV prevention and FP services, with health care providers emphasizing the need for public-private partnerships to enable increased access. Participants cited clinics, pharmacies, and drug shops as specific private-sector outlets that should be involved with integrated HIV prevention and FP service delivery.

“The public sector closes at around 5pm whereby a client may want to access the services at 9pm or 10pm, engaging the private sector would be so important so that we have a win.” (Community leader from Masaka district)

“It’s still through partnerships like I said our District Health Officer we have a WhatsApp group of private and the public facility in-charges and if they have a challenge, they post if the nearby facility for example if our family planning target is 100 and we are seeing 30 people but if the nearby private health facility we have seen 50, then we know that the other 70 is seen by a certain health care provider. So you find that you are looking for the other 20 which hasn’t come to you or to the private health facility. You can even identify different needs, we might not give the private sector the money, drugs but our different IPs [implementing partners] can also come to support the private sector while other can be supporting the public sectors or support both because it’s about increasing the access and if some people cannot access it from public health facility they can still access it from the private.” (Facility in-charge from Masaka district)

Health care providers described the cost of HIV prevention commodities, such as PEP and PrEP, as a limiting factor for their availability in private health facilities, especially given that demand was likely to be low. When thinking about scaling up integrated services, health care providers suggested that these commodities be subsidised in the private sector to make them more affordable, thus increasing availability, uptake, and demand by end users.

#### Voucher system for accessing HIV prevention and FP services

Participants were asked about preferences for a voucher system to access integrated HIV prevention and FP services from private outlets, including where and how vouchers should be issued. Overall, most participants were open to using a voucher system, although some participants were concerned about possible challenges if the voucher process was too complex or time-consuming. Participants had mixed feelings on how vouchers should be accessed—in person via peers, health care providers, at the bank, or online via mobile phone. Overall, getting vouchers from peers was preferred to online systems. A few participants expressed preference for using a mobile money system to access vouchers given added transport costs, although concerns were raised by others about client access to mobile phones. Some participants supported using banks to access vouchers considering safety, timeliness, and confidentiality. A few participants favored using an online system for voucher access as it enabled greater privacy since it only involved the user interacting with the system without any need for explanation or involvement of other people. Other participants said they preferred to get the voucher from the health care providers who deliver HIV prevention and FP services.

Related to the question of who should receive vouchers, the majority of participants suggested giving vouchers to health care providers at government health facilities, but some participants noted that this is not necessary as services at these sites are free. Some suggested providing vouchers to VHT members for easier access by potential clients, since VHT members lived within the community; voucher distribution could also present an opportunity for VHT members to earn an income. Others thought clinics and private drug shops are better suited for the voucher system since they provide fee-based services.

#### Preferred cadre of health workers and sources for delivery of integrated services

The two core factors that shaped preferences for health worker cadre and health care source for the delivery of integrated HIV prevention and FP services were the cost of accessing the service (both payment for the service and transportation) and the ability to ensure privacy and confidentiality. The majority of AGYW participants were of the view that integrated services should be offered by midwives and nurses, as these cadres were considered trained, readily available and professional and therefore able to offer proper counselling and client examinations, adequate guidance, appropriate responses to client questions, and ensure confidentiality.

For the preferred health care source for the delivery of the integrated services, the majority of AGYW preferred government health facilities for offering integrated services because they did not charge for their services. Government health facilities were also described as having qualified health care providers and the necessary storage facilities for HIV prevention and FP commodities. Notably, participants in the co-creation workshop suggested the integrated services should be offered at outpatient departments (OPDs) by nurses and midwives assisted by peer mothers to schedule patients and offer health education talks. Some AGYW suggested that VHT members should deliver integrated services because they were easily accessible in communities, able to spend more time listening to clients, and able to follow up with clients in communities. They said that delivering integrated HIV prevention and FP services through VHT members within communities eliminates or reduces the cost of transport to health facilities.

“I would prefer VHTs to be the ones to get these combined services because they stay in the village and we can easily access these services from them.” (AGYW from Masaka district)

While several AGYW suggested that VHT members could offer integrated services, there were strong concerns related to their ability to keep client information private.

“VHTs would be good because they are near my home but most of them are old and they don’t keep secrets but when I get it from the public service provider it’s safe for me.” (AGYW from Nakasongola district)

Notably, most participants did not support the delivery of the integrated services through private clinic-based providers due to service charges. Similar to VHT members, participants also raised questions about private providers' ability to ensure confidentiality. Most participants highlighted pharmacies as the preferred delivery point if private-sector services were to be considered, as they had greater confidence that client information can be kept confidential and secret, even though some participants noted that pharmacies do not keep client files.

### Use of integrated HIV prevention and FP services

In this section we present the three issues participants highlighted as key for the successful integration of HIV prevention and FP services.

#### Awareness-raising of integrated HIV prevention and FP services

Policymakers, health facility in-charges, health care providers, community leaders, and participants in the co-creation workshop all described demand creation and awareness-raising as critical for uptake of integrated HIV prevention and FP services. Participants highlighted that some HIV prevention services, like PrEP, were not known to many people in the general public, so there is a need for community sensitisation. Community demand creation activities suggested during the co-creation workshop included message dissemination during community meetings, through VHTs/peers, and broader communication methods through megaphones and radios.

“A lot of counselling, a lot of awareness is required both from the client perspective but also the health workers themselves when integrating these services.” (Facility in-charge from Nakasongola district).

In addition, male involvement was recommended during the co-creation workshops as a means of further encouraging acceptance of integrated HIV prevention and FP services. Men could be engaged through targeted outreaches at popular gathering spots, community dialogues, radio health talks, and one-on-one counselling by a peer.

“Men are key stakeholders in this process of designing, piloting, and scaling up the integrated HIV prevention and FP services. The earlier we bring them on board the better the acceptability of the service even among the young women.” (District Health Officer in Nakasongola district)

#### Maintaining confidentiality and privacy

AGYW valued confidentiality and privacy when accessing both HIV prevention and FP services and saw integration of these services as a way of ensuring that their privacy was maintained through engagement with one health care provider. In addition, they felt that making one visit to the health facility to access both services leads to reduction in associated costs as well as minimizes their chances of being seen by other people who might know them.

“To me I say it will be good to use both services and get them once. For example, for me I get family planning from Kyanamukaaka HC IV and I use three thousand shillings and it will save on money for transport and also seeing one provider for both services will save keep privacy of our information because your private information remains with one person.” (AGYW in Masaka district FGD)

Additionally, AGYW felt that seeing one health care provider for HIV prevention and FP services makes it easier to receive counselling, proper guidance, service continuity, and more rapid consultation in case of any challenges. Similarly, health care providers thought clients would be comfortable opening up to one health care provider delivering both HIV prevention and FP services. Other benefits of integrating HIV prevention and FP services raised by health care providers included creating more awareness among AGYW about the existence of HIV prevention services, such as PrEP.

“I think that if we are to offer both family planning and PrEP at the same time, when we are in the community with those AGY, you find that most AGYs know only family planning but when they don’t know what PrEP means. Therefore, when we offer integrated service, it will help the young women to know about PrEP because you find that a young woman needs PrEP but she does not know about it.” (Health care provider in Masaka district FGD)

#### Improved quality of care

Service integration was also seen as improving the quality of care that health workers offered to their clients due to a more holistic understanding of clients' HIV and FP needs, enabling them to determine the best HIV prevention and FP methods for their clients.

“It saves time on both the young women and the health care provider because they are offered at once. It also builds up the friendship between the two parties because you have helped the young woman to get both services at the same time. This means that you are going to know both sides. If you were offering only family planning, it means you will not know things about that person regarding HIV prevention because you are offering one service. But when you offer both services, it means you will be able to get time to go into details of this person on both sides and you also be able to determine the best way to help them.” (Facility in-charge from Masaka district)

#### Perceived challenges of integrated HIV prevention and FP services

AGYW raised concerns that integrating HIV prevention and FP services was likely to lead to delays and prolong the waiting time for clients at the health facility. Instead, they suggested use of age appropriate VHTs or peer mothers to support the providers at OPD with integrated group health education about HIV testing, PrEP and FP services and where resources allow, hiring more providers to make the process smoother.

 “Yes, everything has challenges or effects sometimes you can come for PrEP yet not for family planning and vice versa and they make you wait. Like you can come to pick PrEP and yet you must make a whole line, so at least if they can separate those two delivery points therefore time limit is another challenge.” (AGYW from Nakasongola district)

Some AGYW were skeptical of the possibility of finding one health care provider that was capable of delivering both HIV prevention and FP services, emphasizing that provider training is key. In addition, they felt that one health care provider offering both services could be overwhelming for the provider, thus risk compromising the quality of services they receive. AGYW felt greater task sharing to peer mothers or age appropriate VHTs at the OPDs could mitigate this challenge. There were also concerns that service integration could lead to overcrowding at health facility on the days when these services were offered, which could compromise confidentiality for people who did want to be seen seeking these services by people they know. Participants proposed undertaking infrastructure modifications especially in some facilities where the OPD units are small.

“Me I see it might cause exposure to some people since some of us we are covert users and someone may see me getting both services and goes around and gossips about me.” (AGYW from Masaka district FGD)

It is important to note that there were fears among some AGYW of concurrent side effects with simultaneous use of PrEP and FP. Health education before the service was delivered was mentioned as key to integrated service delivery.

“To me, combining these services is very good but at the same time it is bad because both methods are strong and yet our body systems are weak and even, they react differently. Those PrEP can cause dizziness. I took them but I felt a lot of dizziness and also the family planning has its own effects that it does in the body.” (AGYW from Masaka district)

Health facility in-charges and health care providers described a number of anticipated challenges stemming from integration of HIV prevention and FP services, including (1) lack of required equipment and commodities at the service delivery point, for example, the creatinine test that needed to be carried out before administering PrEP (2); provider knowledge gaps among health care providers regarding new HIV testing methods, such as HIV self-testing, and some FP methods, like the IUD. Similarly, community leaders were sceptical about finding health care providers that were knowledgeable enough to offer both HIV prevention and FP services.

“There is also lack of knowledge in techniques for the new and reformed HIV testing and family planning methods we have. You know that we have OraQuick. There is less knowledge about using it, inserting an IUD, its side effect and giving the knowledge to the client about it. These are the challenges health care provider face.” (Health care provider from Masaka district)

Other client-related challenges included fear of taking an HIV test that was required for PrEP initiation or continuation and withholding of information that could help health care workers make decisions on the best HIV prevention and FP methods for clients. Health care providers also expressed concerns that clients could not afford to pay for integrated services at private facilities, calling for price subsidies on PrEP and FP commodities at private outlets.

### Monitoring and reporting

Health care providers reported using different registers to capture information on HIV prevention and FP services. To maintain high data quality and for easy reporting on PrEP and FP, providers suggested maintaining separate registers for HIV prevention and FP, with registers completed separately by the nurse/midwife. Providers highlighted that training and regular supportive supervision is also key to ensuring data accuracy, timeliness, and completion.

“Since we will continue to report on FP and PrEP separately, we will need the two registers (FP and PrEP) completed separately for quality data and easy reporting. This will be hectic in the beginning but with continuous training and supportive supervision, providers will get used.” (Health care provider from Masaka district)

## Discussion

Integrating HIV prevention initiatives into FP services has the potential to promote access to and increase uptake of HIV prevention and FP services among women. Despite the benefits of integration, HIV prevention and FP services are still being delivered in silos in Uganda. Our interviews with AGYW, health care providers, and policymakers highlight key stakeholder perspectives on enablers and likely barriers to integrating HIV prevention and FP services. Policymakers supported the integration of HIV prevention and FP services and highlighted that current policy guidelines allowed for such integration. Participants suggested that the delivery of HIV prevention and FP services should involve multiple actors at various levels including VHTs, government health facilities, and the private sector [pharmacies, drug shops, non-governmental organizations (NGOs)] to enhance access. Amidst challenges with human resources, respondents suggested task sharing across health worker cadres could resolve some staffing challenges and enable delivery of integrated services. Stockouts of HIV prevention and FP commodities at the health facilities were a potential challenge raised by policymakers and health care providers who recommended joint delivery of sufficient quantities. Integration of HIV prevention and FP services was seen as having several benefits including fostering confidentiality and privacy, allowing for a holistic understanding of clients' FP and HIV status hence improving the quality of care, and creating more awareness among AGYW about the existence of HIV prevention services, such as PrEP. There was concern among health care providers that the introduction of integrated services would come with increased reporting and follow up. Other concerns included delays and prolonged waiting time at the health facility, increased workload for health care providers, overcrowding at the health facility compromising confidentiality, fear of side effects of using HIV prevention and FP services concurrently, lack of required equipment and commodities at the service delivery point, knowledge gaps among health care providers, fear of PrEP reducing the efficacy of FP methods, and clients not being able to afford to pay for integrated services at private facilities. While there is provision for integration in the Ugandan policy and support from policymakers, successful integration will require addressing key concerns raised by participants in human resource and infrastructure, resource management, service delivery and monitoring and reporting.

### Policies

Policymakers supported the integration of HIV prevention and FP services citing benefits of minimizing duplication of services, increasing access to these services, and addressing issues of stigma, particularly related to HIV. Consistent with our study findings, models to integrate FP into HIV care and treatment have been shown to build on the continuity of HIV care and treatment services as a platform from which to reach women and couples living with HIV with additional services to meet their SRH rights and needs (12). Other studies that have involved policymakers highlight the necessity of mobilizing both financial and human resources to facilitate the effective delivery of integrated HIV prevention and FP services. Despite support from policymakers for integration, various policy, structural, and financial barriers pose significant challenges to successful integration efforts. Identified barriers include increased volume of client follow-up and reporting as well as long wait times at health facilities. Separate registers for HIV prevention and FP services also creates logistical obstacles to service delivery (19). Cost implications of integrating services can be challenging, as evidenced in the evaluation of a cluster randomized trial in Kenya, which assessed the cost of implementing integrated services (20). Financial considerations and resource constraints can hinder the effective implementation of integrated FP and HIV services, impacting the availability and quality of care provided to AGYW. Addressing these barriers is crucial to ensuring that AGYW have equitable access to comprehensive and integrated SRH services.

### Human resources and infrastructure

Participants in this study noted a need for increased staffing levels and task sharing across cadres to enable successful delivery of integrated HIV prevention and FP services. This aligns with findings from previous studies that underscore the importance of training, increasing staffing, and provision of job aids and guidelines to guide implementation (21). Research among service providers highlighted the challenges in delivering integrated services including lack of space, time, insufficient staff, inadequate training, and commodity shortages (22). Consistent with our findings, other studies in sub-Saharan Africa highlight the importance of provider training, ongoing mentorship, and capacity building for ancillary staff for enabling successful integration (23). Participants in this study also identified limited space as a challenge for delivering integrated services in a confidential and friendly manner. These findings are consistent with other studies, for example, in Ethiopia where a reported lack of space, time constraints, and insufficient staff can impact the delivery of youth-friendly integrated services (24). There is an urgent need for comprehensive strategies to address staffing, training, and infrastructure limitations in health care facilities if the integration of these services is to be successful.

### Resource management

Stockouts of HIV prevention and FP commodities at health facilities were cited as a key challenge for integration in our study, especially in private health facilities where PrEP was reported to not be available. Both policymakers and health care providers emphasized the need to ensure that there was a consistent supply of commodities at the health facilities. In a study conducted in Kenya, many providers reported experiencing a shortage of contraceptive commodities, which affected the impact of integrated services (17). Studies have demonstrated that medication shortages affect how people seek medical attention, highlighting the negative effects of stockouts on service provision (25). In addition, health care providers and health facilities in charge believed that to enable the successful delivery of integrated HIV prevention and FP services, commodities for both services needed to be delivered jointly and in sufficient quantities. The recent changes in global policy supporting major donors' integration of SRH/HIV services and the emergence of new bilateral donor agreements to jointly fund HIV commodities and contraceptives further support the idea of delivering both HIV prevention and FP services commodities jointly and in sufficient quantities (26). Our study findings highlight the necessity of tackling stockouts and guaranteeing a steady supply of HIV prevention and FP supplies to facilitate the efficient provision of integrated services.

### Service delivery

Health care providers in our study suggested that VHTs be engaged to carry out integrated community sensitization and service delivery, pointing out that some VHT members had already been trained to deliver PrEP and injectable contraceptive DMPA-SC (Sayana Press) in communities. A study conducted in Uganda showed that involving trained VHTs in the delivery of services made it easier for women to access injectable FP methods, which improved their use as indicated by a rise in the unmet need for contraception (27). The significance of providing HIV prevention and FP supplies to government health facilities to guarantee availability of consumables, thus increasing user accessibility and improving provision of comprehensive care, has been emphasized in previous studies (28, 29). Integration of services and the assurance of consumable availability emerge as critical strategies for health care facilities to enhance the provision of comprehensive care to young girls seeking HIV prevention and FP services.

Community outreaches were cited by health workers as additional channels for extending the reach of integrated HIV prevention and FP services to end users. Outreach programs have been emphasized as essential to enhancing the connection between patients with HIV diagnosis and care, highlighting the significance of fortifying health systems linkages with communities to foster partnerships and improve care accessibility (30). By delivering services in the community, outreach programs can reach individuals who might find it difficult to access care in facilities. Numerous studies have demonstrated that peer-led community-based outreach programs have proven particularly successful in reaching vulnerable populations, including people who use drugs, with HIV and harm reduction therapies (31). Strengthening the relationships that health systems have with local communities has the benefit of improving access to care, which can ultimately lead to the provision of more thorough and equitable services.

While the private sector was cited by our study participants as a crucial strategy for ensuring last-mile delivery of integrated HIV prevention and FP services, some health workers raised concerns on quality of counselling on PrEP use and following all required steps and clinical examinations before enrolling clients on and/or dispensing PrEP. Health care providers described the cost of HIV prevention commodities, such as PrEP and post-exposure prophylaxis, as a limiting factor for their availability in private health facilities. Similarly, some studies have demonstrated that the use of the private sector for HIV testing within countries is frequently associated with wealth, suggesting that access may differ depending on socioeconomic status (32). The necessity for public-private partnerships (PPPs) was highlighted by the health care workers in our study as a means of facilitating greater access to HIV prevention and FP services. While some research emphasizes the need for enhanced infrastructure and service quality and public and private facilities, respectively, to facilitate PPPs for increased FP utilization and better health outcomes (33), other research highlights the gap in evidence linking PPPs to health outcomes (34). Moreover, PPPs in public health have drawbacks despite their possible advantages, such as issues with inequity and inefficiency (35).

Overall, most participants were open to using a voucher system to access integrated FP and PrEP services preferably received from peers (health care providers or VHTs) rather than online systems. Using voucher programs has been shown to be an effective way to increase marginalized groups' access to FP services (36). Voucher interventions have been proven to improve access to care for disadvantaged and impoverished people, especially when they are targeted and implemented in a pro-poor strategy (37). Sensitization campaigns are important in health care institutions and communities since the effectiveness of voucher programs may be impacted by the level of awareness and use of vouchers by health care professionals and clients (38).

The majority of AGYW participants in our study believed that midwives and nurses should offer integrated services. Research studies have shown that in addition to their routine responsibilities of delivering maternal health services, nurses and midwives are increasingly being requested to perform HIV prevention-related tasks, such as the prevention of mother-to-child transmission of HIV (39). Training in FP and HIV management improves nurses' integration of HIV prevention and SRH (40). AGYW participants' endorsement of integrated services highlights the critical role played by nurses and midwives in providing comprehensive care in government health institutions. The knowledge and experience of nurses and midwives would enhance their ability to integrate HIV prevention initiatives smoothly and effectively. While VHTs were suggested as an option for offering integrated services, there were concerns regarding their ability to maintain confidentiality. Concerns about privacy and the quality of care are obstacles to using the FP services offered by VHTs; for example, one research study showed 60% of participants reported concerns that their privacy would be violated by VHTs (41). Implementing comprehensive care delivery models could help relieve worries about privacy and confidentiality (42).

### Use of integrated HIV prevention and FP services

The need to create awareness regarding the availability of integrated HIV prevention and FP services was raised by policymakers, health facility in-charges, health care providers, and community leaders in our study as a means of leading to greater HIV prevention and FP uptake. Participants highlighted that some HIV prevention services like PrEP were not known to many people in the general public and there would be a need to engage in thorough sensitization of communities. A study conducted in Uganda showed that AGYW with high vulnerability to HIV had little knowledge of injectable and oral PrEP (43). Many factors, such as restricted information on and low awareness of HIV prevention strategies, social stigma, and lack of social support, prevent AGYW from using PrEP (44). To reduce HIV incidence, research has also shown how critical it is for AGYW to increase their education, risk perception, awareness, and adoption of PrEP (45). A higher rate of testing for HIV and sexually transmitted infections among AGYW has been linked to awareness of local HIV prevention programs (46). Successful integration of HIV prevention and FP services must be backed by the support and commitment of policymakers. We also acknowledge that more robust social behavior change activities to address social norms and beliefs around sexuality and deeper work to systematically assess and address persistent stigma-related barriers impeding HIV prevention and FP use is critically needed to enhance PrEP uptake and effective use.

Our study participants viewed the integration of HIV prevention and FP services as a way of ensuring that their privacy was maintained. Although some studies have shown the advantages of integrated FP and HIV services, preserving confidentiality in integrated models continues to be difficult (47). Integration of these services was also seen as improving the quality of care that health workers offered to their clients as they would have a holistic understanding of clients' FP and HIV status, enabling them to determine the best HIV prevention and FP method for their clients. A study conducted in Tanzania showed that integrating FP into HIV prevention services through a facilitated referral strategy would contribute to possible improvements in the quality of care (48). However, research studies carried out in Malawi and Tanzania, reported that there was no statistically significant difference in the quality of FP services between HIV-integrated and non-integrated facilities (28). This discrepancy highlights the complexity of integration efforts and suggests the need for further investigation into the factors influencing care quality outcomes in integrated settings.

There was concern among health care providers in our study that the workload of health workers would increase following service integration due to increased reporting and follow-up. Consistent with our findings, research studies have reported that health workers face challenges as a result of the integration of various services, including an increase in stress and workload (49, 50). For example, following the integration of HIV and postnatal treatments, health workers in Kenya reported professional stress associated with increased workload and restricted time for patient counselling (50). These concerns highlight the importance of carefully assessing the impact of service integration on health care providers and implementing strategies to mitigate potential challenges. AGYW raised concerns that integrating HIV prevention and FP services was likely to lead to delays and prolong the waiting time for clients at the health facility. A research study on the integration of HIV and cervical cancer screening programs in Uganda showed community concerns regarding possible delays at health facilities as a result of integration, which could wear out health workers and women who are seeking services (51). These findings suggest that service integration may have an effect on waiting times and the burden of health workers. These concerns extend beyond mere inconveniences because long waiting times can burden health workers and AGYW seeking FP and HIV preventive services. The community's viewpoints and any possible effects on service delivery should be considered while implementing integration projects.

Some AGYW were skeptical of the possibility of finding one health care provider that was capable of delivering both HIV prevention and FP services. Research has shown that FP providers have been identified as major players in providing PrEP to women, demonstrating the changing roles of health care providers in HIV prevention (52). In addition, they felt that one health care provider offering both services would be overwhelming for the provider, which would risk compromising the quality of services they receive. Research has shown that integrating HIV care into existing services, such as prenatal clinics, can be challenging for health workers, resulting in more workload and the need for additional training (53). When health workers are overworked by being in charge of providing many services, the quality of care in integrated settings may be compromised. This is likely to result in a patient-provider disconnect, hence affecting the overall quality of care offered (54). It is key to address these concerns to ensure that integration efforts effectively enhance service delivery and maintain high standards of care.

There were also concerns that service integration would result in the health facility being overcrowded on the days when these services were offered, which could compromise confidentiality for people who did not want to be seen seeking these services. Studies show that the integration of services increases the client volume or provider workloads and is likely to lower the quality of care (40, 55). It is important to adopt a user-centered design process that supports the integration of the HIV prevention and FP services by focusing on promoting privacy and confidentiality and highlighting the key role of the peer (56). By prioritizing these aspects, integration efforts can better meet the needs of clients while maintaining high standards of care.

There were fears among some AGYW that receiving both HIV prevention and FP methods would lead to clients encountering concurrent side effects from both products. Providing women with comprehensive information on the effectiveness, side effects, and follow-up required for the FP method of their choice during the HIV-tailored FP counselling for women is crucial (57). By integrating such comprehensive counselling practices, health care providers can minimize the side effects reported by AGYW due to integrated HIV prevention and FP services. Our study participants highlighted the challenge of a lack of required equipment and commodities at service delivery points (for example, supplies for creatinine testing required before administering PrEP). Shortage in commodities has a detrimental impact on the responsiveness of services in the integrated delivery of FP services (58). The need to increase efforts to overcome supply-side constraints, particularly associated with commodity shortages when offering integrated HIV prevention and FP services has been emphasized (18). Integrated HIV prevention and FP service providers should intensify efforts to overcome supply-side constraints.

### Limitations

This study had several limitations. First, the study was only conducted in two districts of Uganda due to operational constraints. To ensure a more representative sampling, to the extent possible, the study team worked with the Uganda MOH and TAG members to carefully select districts to ensure geographic and service coverage diversity. As such, the two selected districts provide a comprehensive picture of insights from an urban setting with high awareness and experience with FP and HIV services (Masaka) and a rural setting with less experience with such services (Nakasongola).

A second limitation was data collection methods used by AGYW peer researchers to gather insights and data from health care providers, managers, and policymakers. To mitigate this limitation, AGYW peer researchers were trained in research ethics, human-centered design, and effective communication skills to facilitate interviews and mobilize participants; each peer researcher was also linked to an experienced research assistant for ongoing coaching and mentoring throughout the study. Additionally, to ensure high quality data collection, AGYW peer researchers received comprehensive training on qualitative data collection techniques, data cleaning, and transcription.

## Conclusions

The findings presented in this paper contribute to the growing body of knowledge on integration of HIV prevention and FP services in low-income settings. Integration of HIV prevention and FP services has been described as complex as its success requires addressing various health system factors around human resources, infrastructure, service delivery and monitoring and reporting. Our study highlights enablers and barriers of integration of these services presented under five domains: policy, human resources and infrastructure, resource management, service delivery, and monitoring and reporting. While the integration of HIV prevention and FP services presents various benefits of increasing access to and uptake of these services, its successful implementation greatly depends on addressing challenges with human resources, infrastructure, stock management, and monitoring and reporting as well as a more robust and continuing focus on addressing social and gender norms and drivers of stigma impeding HIV prevention and FP use. Advancing integrated HIV prevention and FP models in Uganda, and across sub-Saharan Africa, is not only critical to enhancing service access and continuity through promotion of integrated, person-centered delivery models but to also create future platforms for delivery of dual prevention products for HIV and unintended pregnancies currently in the development pipeline, such as the Dual Prevention Pill (16). Understanding and introducing feasible and acceptable integrated HIV prevention and FP models now would allow to more rapid roll out and scale up of these critical prevention products as they enter the market.

## Data Availability

The raw data supporting the conclusions of this article will be made available by the authors, without undue reservation.
